# Partial dark-field microscopy for investigating domain structures of double-layer microsphere film

**DOI:** 10.1038/srep10157

**Published:** 2015-05-11

**Authors:** Joon Heon Kim, Jung Su Park

**Affiliations:** 1 Advanced Photonics Research Institute, Gwangju Institute of Science and Technology, Gwangju 500-712, Republic of Korea

## Abstract

A lateral dislocation in a double-layer microsphere film is very difficult to identify because the constituent domains have the same two-dimensional crystalline orientation. Orientation-sensitive optical techniques cannot resolve this issue. Here, we demonstrate that partial dark-field (pDF) optical microscopy can be very effective in identifying this type of domain boundary and dislocation of a close-packed microsphere double-layer. Using the hexagonal symmetry of the close-packed microsphere film and the light-focusing property of microspheres, the partially blocked dark-field condenser can provide much higher contrast than other optical microscopy modes can in identifying the laterally dislocated domains. The former can also distinguish domains with different crystalline orientation by rotating the pDF stop. The simplicity of the pDF mode will make it an ideal tool for the structural study of close-packed double-layer microsphere films.

Close-packed microsphere films have been intensively studied in recent years because of their possible application as templates for nanostructures or photonic crystal structures^1^,^2^,^3^,^4^,^5^. Because it is not easy to obtain a large single-domain microsphere film, it is important to identify domain boundaries and defects in the prepared microsphere film. One of the simplest methods to identify the crystalline structure of the microsphere film is the identification of the position of each microsphere by optical microscopy. However, the immediate identification of domain boundaries in a large area using the common bright-field (BF) mode of optical microscopy is not very easy because of the low contrast.

For a monolayer microsphere film, the principal difference among domains is mostly related to the different two-dimensional crystalline orientations. Therefore, an orientation-sensitive technique to distinguish domains with different crystalline orientations can be very helpful to achieve increased visibility of domain boundaries. In this sense, previously, we have suggested polarized microscopy (PM) as an ideal tool for the fast examination of a microsphere monolayer because PM is much more effective than common BF microscopy in rapidly identifying the crystalline orientation of domains^6^.

However, for a double-layer microsphere film, which has also been frequently used as a template for more sophisticated nanostructures^7^,^8^,^9^,^10^, another type of important defect can exist even between domains with the same two-dimensional crystalline orientation: a dislocation (Fig. 1). Because upper-layer microspheres should be located on the hollows formed by three contacting microspheres of the lower layer in the close-packed conformation, two different stacking conformations of the same two-dimensional crystalline orientation are possible (Fig. 1). Both domains have a three-fold symmetry invariant under rotations of 120° and can be mapped into each other by applying a rotation of 60°. Although the domains are otherwise indistinguishable, they cannot be merged into a single domain when they meet together, which generates a dislocation in the upper layer.

This type of domain boundary cannot be easily identified, particularly for small microspheres, because the main difference between these two domains is not the crystalline orientation but the position of the microspheres. Therefore, an orientation-sensitive technique, such as the previously suggested PM mode^6^, cannot effectively distinguish these two domains because it is not sensitive to the lateral position of the microspheres. The overlap of images from each layer also makes it difficult to directly apply this technique for the double layer film. When we want to focus on the upper layer, light from the lower layer hinders the clear distinction of the different domains in the upper layer. Although the gap between domains can be partially visualized at high magnification, this visualization is not easy, particularly for a film of small microspheres, because of the low contrast.

In this paper, we will demonstrate that a slight modification of dark-field (DF) optical microscopy, which we call the partial dark-field (pDF) mode, can be very helpful in identifying this type of domain boundaries in the double layer. Using this technique, even a slight dislocation among domains with the same orientation can be easily identified by the greatly enhanced contrast. This increased contrast is primarily due to the symmetry matching of the modified DF stop to the hexagonally symmetric close-packed microsphere film and the characteristic ring-like bright region only at the surface of the microsphere in the DF mode induced by the focusing of light through the lens effect of microspheres. The principle of this technique and applications for different sizes of microsphere double-layers will be presented.

## Results and discussion

Figure 2 presents images of a polystyrene microsphere in the BF, DF, and partial DF (pDF) modes. In the BF mode, the microsphere appears as a dark ring with a bright background because much of the scattered light is not detected by the objective lens (Fig. 2a). On the contrary, the microsphere appears as a bright ring with a dark background in the DF mode (Fig. 2b) because only the deflected light can be captured by the microscope objective.

In contrast to the general explanation for the DF image-forming mechanism, this bright ring does not originate from the directly scattered (or reflected) light at the microsphere surface. Instead, it originates from the focused light due to the lens effect of the microsphere on the obliquely incident light passing through it (Fig. 2d). It is known that a localized nanoscale photonic jet can be generated at the shadow-side surface of microsphere when it is illuminated by light^11^,^12^,^13^. Depending on the size and refractive index of the microsphere, this focused light can be formed very near the surface of the microsphere. The focusing of light by the microsphere can deflect the initial direction of light such that part of the focused light can be directed to the objective lens. In addition, part of the focused light can be scattered at the microsphere surface when the focal point is very near the microsphere surface, and this scattered light can also be detected by the objective.

This explanation can be clearly demonstrated using the pDF mode (Fig. 2c). In this mode, only some parts of DF illumination are allowed to be incident on the sample by blocking parts of the annular ring of the common DF condenser using a patterned stop (see Supplementary Information, Fig. S1). In Fig. 2c, a modified DF stop consisting of three symmetric open-arcs, each of which spans an angle of 60°, was used for the pDF illumination. Notably, the bright arcs on the microsphere surface in this image are located on the opposite sides of the incident DF illumination light (marked by arrows). This result clearly demonstrates that the bright arcs in the DF mode mostly originate from the focusing of light through the microsphere rather than the directly scattered (or reflected) light at the microsphere surface. The images of the microsphere at different focal planes further support this idea (Fig. 2e). In these consecutive images, the position of the bright arc moves outwards as the focal plane of the objective lens moves downward, which indicates that the bright arc should not be the directly scattered light at the microsphere surface. If the bright arc is caused by the direct scattering of the incident light at the microsphere surface, the point at which the light direction can be deflected to the direction of the objective should follow the contour of the microsphere surface. Therefore, the position of the bright arc should move inwards as the objective focal plane moves downward. In contrast, if the deflection of light is caused by the focusing of light through the microsphere, the position of light deflection should be located at the focal point in the space, and the propagation of light should move outwards as the objective focal plane moves downward, which is consistent with the images in Fig. 2e.

Because the hexagonally close-packed microsphere film has an angular symmetry of 60°, the application of a 120° angular symmetric pDF stop (as shown in Fig. 2c) can result in interesting images depending on the relative orientation of the two-dimensional crystalline axis and the pDF stop. By carefully matching the crystalline axis of the microsphere monolayer and the orientation of the pDF stop, we can obtain an image of bright triangles at the hollow of three contacting microspheres (Fig. 3b). Half of the total hollows appear as bright triangles, while the other half are dark. By rotating the pDF stop by 60°, we can obtain an image of bright reversed triangles at the previously dark hollows, whereas the previously bright hollows appear dark in this configuration (Fig. 3d). At the intermediate angle of 30°, the bright arcs of the nearest microspheres are positioned successively to each other (Fig. 3c), which provides a similar image to that of the full DF image (Fig. 3a).

These characteristic patterns in the pDF image are more distinct for the smaller microsphere (a diameter of 2 μm) film (Fig. 4a–d). Here, the bright rings in the full DF image (Fig. 4a) and pDF image with a relative angle of 30° (Fig. 4c) appear almost connected, and the bright parts of the pDF images with a relative angle of 0° or 60° show more triangular shapes (Fig. 4b,d).

The finding that the positions of bright triangles are alternately changed with a 60° rotation of the pDF stop can be effectively used in distinguishing the lateral position of the upper-layer microsphere placed on top of the hollows of the lower microsphere layer (Fig. 4e–h). The light through the lower layer can act as a light source to shine on the upper-layer microspheres. Therefore, a microsphere located on top of the bright hollow of the lower layer can be imaged as a bright sphere, whereas that on top of a dark hollow appears dark (Fig. 4f,h). This finding indicates that microspheres just slightly dislocated (by approximately the radius of a microsphere) from the regular position of the upper-layer microsphere domain can be clearly distinguished by the large contrast. For the intermediate angle of 30° (Fig. 4g), all of the upper-layer microspheres appear equally bright, similar to those in the full DF image (Fig. 4e).

Figure 5 demonstrates how this property can be used to effectively identify the domain structures of the upper-layer microspheres. Here, domains A and B both have the same two-dimensional crystalline orientation; however, their upper layers are slightly shifted from each other, forming a dislocation between them. Because a small gap exists between the domains, dislocations at the domain boundary can be visualized as thin dark lines in the full DF image; however, the contrast remains poor (Fig. 5a). In particular, the identification of these dislocations becomes more difficult for the large-area image (Fig. 6a). However, two similar domains can be clearly distinguished by the large contrast in the pDF images (Figs. 5b-d, 6e-h) making the identification of domain boundaries much easier.

The regions of different two-dimensional crystalline orientations appear as gray regions with intermediate brightness (Fig. 6e–h). The differently oriented domains in the upper layer are caused by the differently oriented domains of the lower layer because the crystalline orientation of the upper layer and lower layer in the double-layer microsphere film should be the same in the close-packed conformation. The regions with maximum brightness and minimum brightness have the same crystalline orientation except that their array positions are laterally shifted from each other by a minute amount. By rotating the pDF stop, these gray regions can also become the brightest or darkest regions at some specific angle. By measuring this angle, we can identify the crystalline orientation of the specific domains.

This technique can be well applied for other sizes of microspheres (down to a diameter of 0.5 μm at least) as long as they form a hexagonally close-packed double-layer film (Fig. 6f–h). The advantage of this technique is more prominent for smaller microspheres, for which the identification of the exact position of every microsphere becomes more difficult. For example, even when the exact position of each microsphere with a 0.75- or 0.5 μm diameter is not clearly identified in the full DF image, the pDF method successfully distinguishes the dislocated domains by the good contrast (Fig. 6g,h).

Because the primary goal is to distinguish different domains rather than to identify individual microspheres, the applicability of this technique to smaller microspheres can be expected to be relatively less affected by the diffraction-limited optical resolution for the size of the microsphere. However, for very small microspheres, for which the scattering of light can be more dominant than the focusing of light, the pDF technique will not work well because light cannot be efficiently focused on the hollows by the lens effect of the lower-layer microspheres. Moreover, the focusing volume can be larger than the distance between nearest hollows such that the rotation of the pDF stop cannot alternatively illuminate different hollows. Therefore, the pDF technique should also be affected by the diffraction-limited optical resolution. In our experiment, the pDF technique did not work well for microspheres smaller than 300 nm.

**Figure 1:**
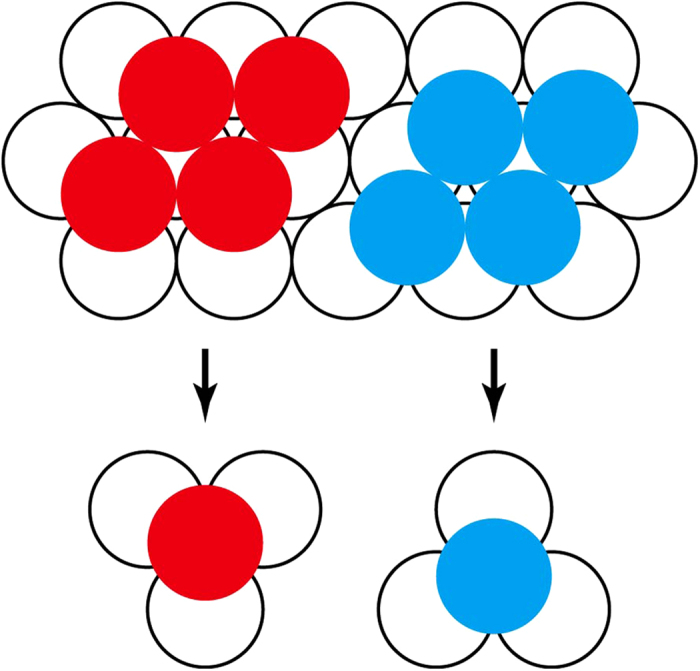
Schematic diagram of the domain boundary of the double-layer microsphere film caused by the dislocation of the 2^nd^ layer microspheres.

**Figure 2:**
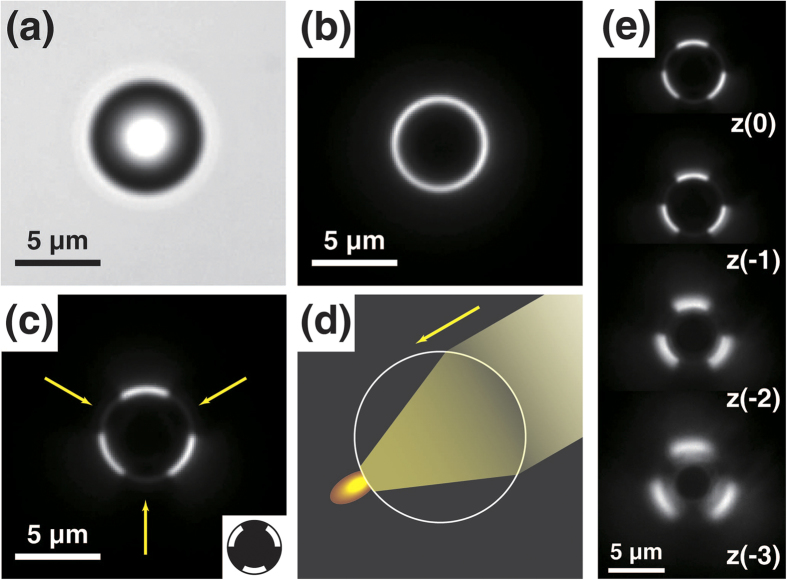
(**a**) BF, (**b**) full DF, and (**c**) pDF images of a single polystyrene microsphere with a diameter of 6 μm. The inset in (**c**) shows the shape of the pDF stop used in this experiment. Light can pass through only three symmetric open arcs. The yellow arrows indicate the light directions incident on the microsphere. (**d**) Schematic diagram: obliquely incident light by the DF condenser can be focused on the opposite side of the microsphere. (**e**) Images of a microsphere with a consecutive change of the focal plane by a step of 1 μm. With reference to the focal plane of the clearest image (*z* = 0), decreasing *z* indicates that the focal plane is farther from the light source.

**Figure 3:**
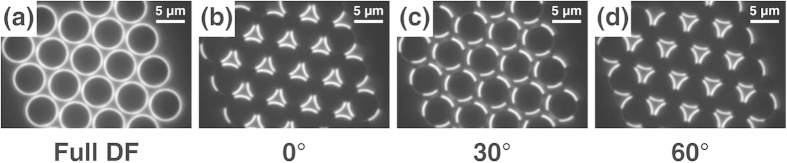
(**a**) Full DF and (**b**-**d**) pDF images of the 6 μm polystyrene microsphere monolayer with a pDF stop rotation angle of 0°, 30°, and 60°. The indicated rotation angles are the relative values with reference to the angle of the pDF stop for the image (**b**).

**Figure 4:**
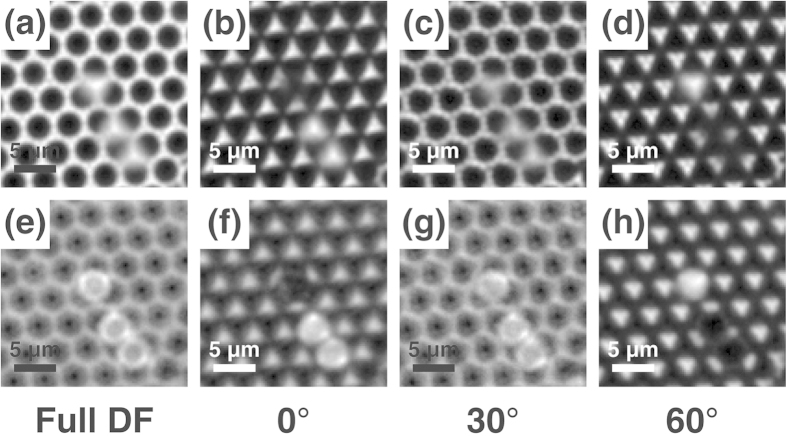
Full DF and pDF images of the 2 μm silica microsphere double-layer with a pDF stop rotation angle of 0°, 30°, and 60°. (**a**-**d**) Images focused on the lower layer. (**e**-**f**) Images focused on the three microspheres of the upper layer.

**Figure 5:**
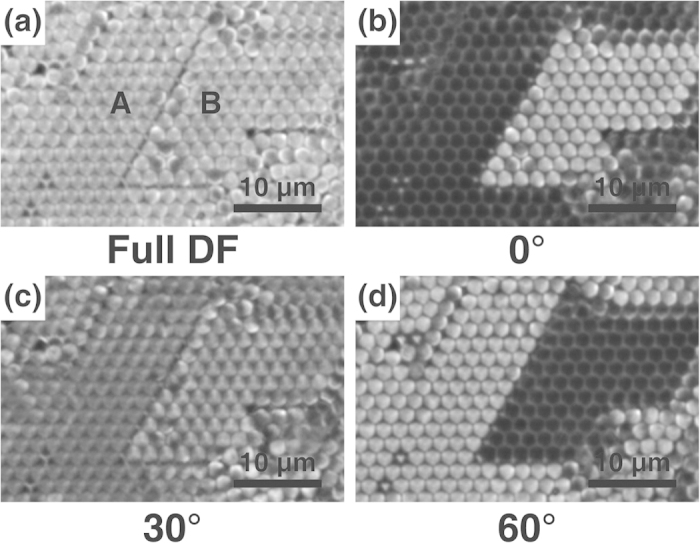
(**a**) Full DF and (b-d) pDF images focused on the upper layer of the 2 μm silica microsphere double-layer with a pDF stop rotation angle of 0°, 30°, and 60°. Domains A and B are slightly dislocated from each other such as in the schematic diagram in Fig. 1.

**Figure 6:**
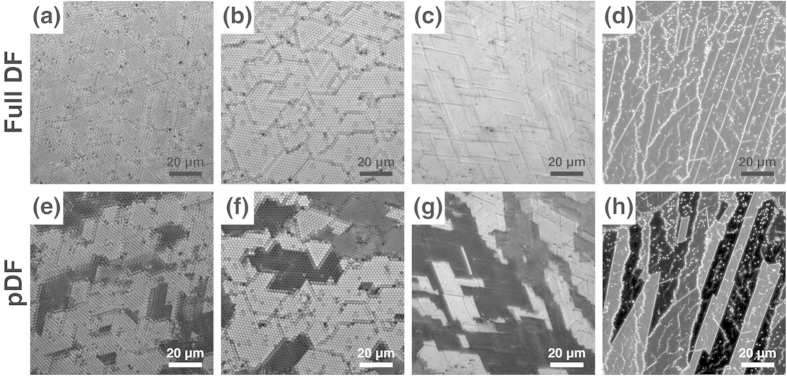
Large-area full DF and pDF images of the double-layer film consisting of various sizes of microspheres: (**a**,**e**) 2 μm silica, (**b**,**f**) 3 μm polystyrene, (**c**,**g**) 0.75 μm polystyrene, and (**d**,**h**) 0.5 μm polystyrene. Light sources: a 405 nm LED for (**a**-**c**,**e**-**g**) and a halogen lamp for (**d**,**h**). See Supplementary Information.

On the other hand, when the microspheres of the upper layer have a different size than the microspheres of the lower layer, the pDF method is expected to work well. As long as the microspheres of the upper layer are sufficiently large to have a dislocation such as that described in Fig. 1, the pDF method will be able to distinguish different domains with large contrast using the same principle as that used for the single-sized microspheres (see Supplementary Information).

The pDF method has an additional advantage that it can be applied with a much weaker light source compared with the previously suggested PM method^6^. In a PM image, a very strong light source must be used to obtain an image with sufficient brightness because the depolarization effect at the microsphere surface is not very strong. In contrast, the bright arcs in the pDF image result from focusing of light such that even a weak light source can generate a very bright image. In addition, even if we used a 405 nm LED to obtain most of the pDF images presented in this paper, the white light from a commonly available halogen lamp also provides sufficient contrast (see Supplementary Information).

In conclusion, we demonstrated that the pDF mode is a very effective technique for the visualization of domain boundaries and dislocations in close-packed double-layer microsphere films. Compared with other optical microscopic modes, this mode yields very large contrast between even very similar crystalline domains that have the same two-dimensional crystalline orientation but are slightly dislocated from each other. These domains appear as the brightest and darkest regions in each pDF image at a specific orientation of the pDF stop. By rotating the pDF stop, we can continuously change the brightness of the domains depending on their crystalline orientation. The simplicity of changing from the common full DF to the pDF mode simply with the addition of the patterned stop is also advantageous for practical application. We believe that the suggested pDF technique will extend the versatility of optical DF microscopy in the study of close-packed microsphere films.

## Methods

A dried film of polystyrene or silica microspheres with various diameters (Polysciences) was formed on cover glass and was then placed on the sample stage of an inverted microscope (OLYMPUS IX71) with the microsphere film side oriented downwards (Fig. S1). A dry dark-field condenser (U-DCD: NA 0.8-0.92) with a 405 nm LED or a halogen lamp as a light source and a long-working-distance 60x objective (NA 0.7) were used for the transmission DF mode. A patterned stop with three symmetric open apertures, each of which span an angle of 60°, was added to the U-DCD condenser for the partial DF illumination. The images were captured using a sCMOS camera (Andor, NEO).

## Additional Information

**How to cite this article**: Kim, J. H. and Park, J. S. Partial dark-field microscopy for investigating domain structures of double-layer microsphere film. *Sci. Rep.* **5**, 10157; doi: 10.1038/srep10157 (2015).

## Supplementary Material

Supplementary Information
