# Supplementary material for: Partial dark-field microscopy for investigating domain structures of double-layer microsphere film
Source: Sci Rep. 2015 May 11;5:10157. doi: 10.1038/srep10157 (PMC4426674; doi:10.1038/srep10157)

Supplementary Information

Partial dark-field microscopy for investigating domain structures of double-layer microsphere film

Joon Heon Kim* and Jung Su Park

Advanced Photonics Research Institute, Gwangju Institute of Science and Technology, Gwangju 500-712, Republic of Korea

* Corresponding author: joonhkim@gist.ac.kr

(1) Experimental setup

Figure S1. A rotation mount holding a pDF stop is added on a commercial dark-field condenser (Olympus, U-DCD). For the specific crystalline orientation of the domain, we can find a specific rotation angle of the pDF stop showing the maximum or minimum brightness of that domain. We set this angle as 0°, and the other angles are defined as relative values, considering this angle as the reference angle. For the full DF image, the pDF stop can be removed from the light path.

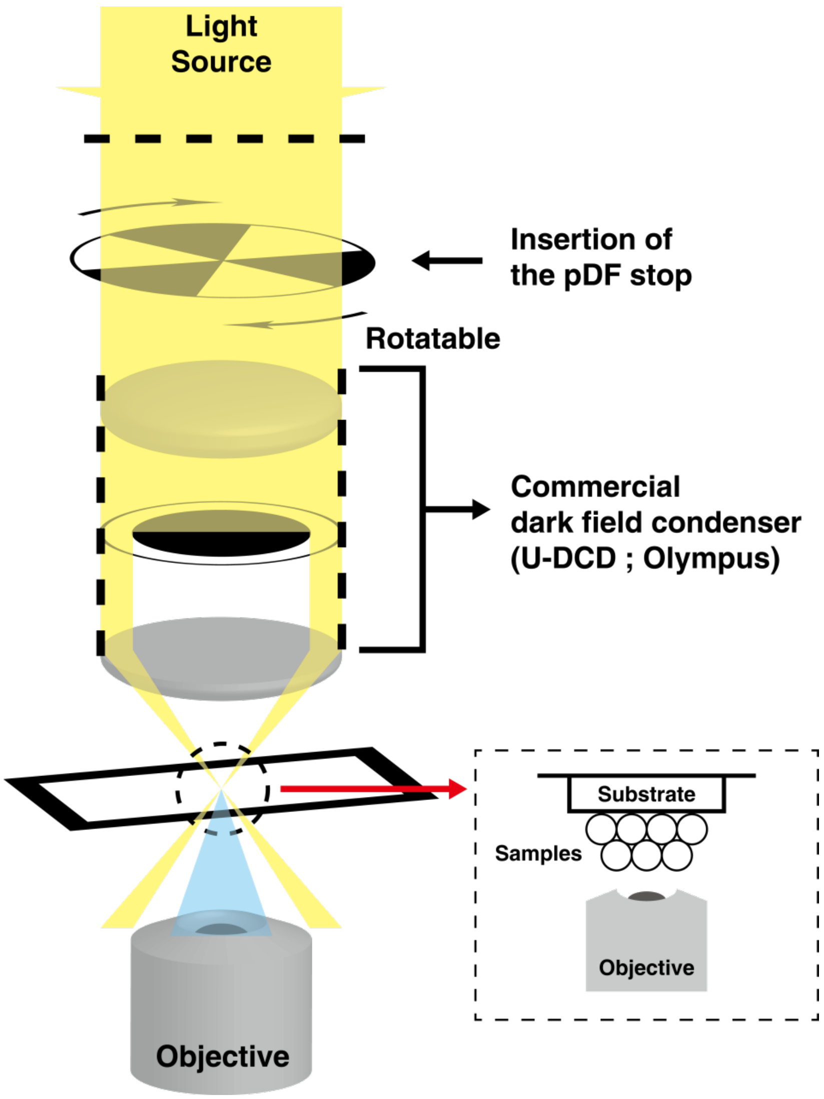

(2) Light sources for the pDF images

We used a 405-nm LED instead of white light from a halogen lamp to obtain most of the images in this manuscript to minimize the possible chromatic aberrations. However, for the case of microspheres larger than 0.75 μm, both a 405-nm LED and a halogen lamp produced a sufficiently good contrast in the pDF images.

In contrast, for the case of 0.5-μm microspheres (Figs. 6d & 6h in the manuscript), a 405-nm LED did not provide sufficient contrast in the pDF mode compared with white light from the halogen lamp. When a film of this size of microspheres was observed using white light, a specific wavelength of visible light (blue light in this case) was dominant in the acquired image. Therefore, for a small microsphere, the diameter of which is comparable to the wavelength of visible light, either the specific wavelength of light matching the microsphere size or white light should be used to obtain maximum contrast in the pDF image.

(3) Applicability to the double-layer of different sized microspheres

There are many applications in which the microspheres of the upper layer have a different size than the microspheres of the lower layer.

When the diameter (ϕ_A) of the upper-layer microsphere is smaller than $\frac{1}{\sqrt{3}}$ times the diameter (ϕ_B) of the lower-layer microsphere, every hollow of the lower layer can be occupied by the upper-layer microspheres because there should be no overlap between the upper-layer microspheres on the nearest-neighbor hollows of the lower layer (Fig. S2). In this case, a dislocation such as that described in Fig. 1 of the manuscript cannot exist, and most defects of

the film may originate from the 2D crystalline orientation mismatch. Therefore, the pDF method would not be necessary for this case.

When ϕ_A is larger than $\frac{1}{\sqrt{3}}$ times ϕ_B, only half of the hollows of the lower layer can be occupied by the upper-layer microspheres, similar to the case of the single-sized microspheres, because of the geometrical constraint between the upper-layer microspheres on the nearest-neighbor hollows of the lower layer. In this case, we expect that the pDF method can effectively distinguish the dislocation with a large contrast, similar to the case of the single-sized microspheres.

Figure S3 shows a pDF image of a binary film consisting of 2 um Silica beads as the lower layer and 1 um PS beads as the upper layer. Even though we could not fabricate a close-packed double-layer film, this figure clearly shows that the pDF method can alternately illuminate the upper-layer beads depending on their positions.

Figure S2.

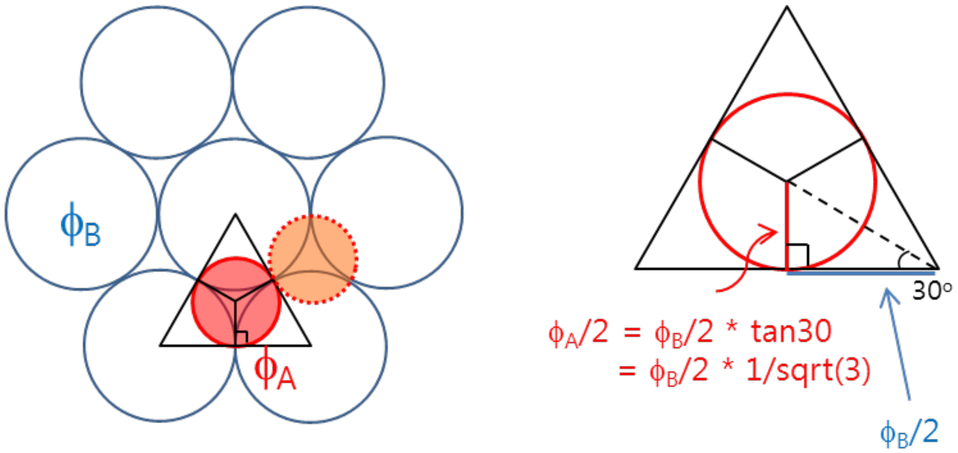

Figure S3. A rotation of a pDF stop by 60° can alternately illuminate the upper-layer beads depending on their positions. (For example, beads at the red arrows *vs.* beads at the green arrows)

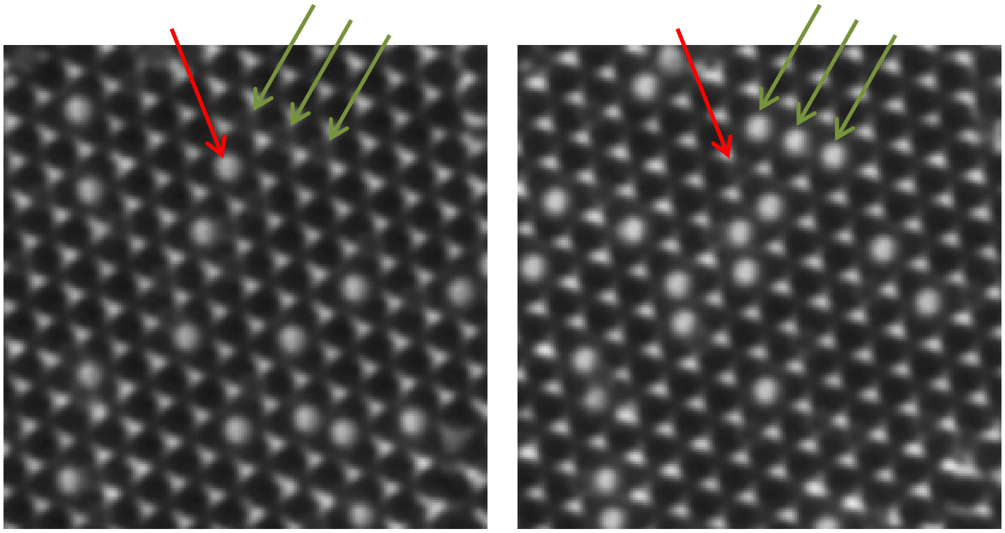

Supplement: Supplementary Information [file srep10157-s1.pdf]
